# Efficacy of Cilnidipine (L/N-type Calcium Channel Blocker) in Treatment of Hypertension: A Meta-Analysis of Randomized and Non-randomized Controlled Trials

**DOI:** 10.7759/cureus.19822

**Published:** 2021-11-22

**Authors:** Rabindra Nath Chakraborty, Deepak Langade, Shyam More, Vaibhav Revandkar, Ashish Birla

**Affiliations:** 1 Cardiology, Medica Superspecialty Hospitals, Kolkata, IND; 2 Pharmacology, School of Medicine, D Y Patil University, Navi Mumbai, IND; 3 Community Medicine, School of Medicine, D Y Patil University, Navi Mumbai, IND; 4 Medical Affairs, J. B. Chemicals and Pharmaceutical Limited, Mumbai, IND

**Keywords:** rct, cilnidipine, l/n-type calcium channel, hypertension, calcium channel blocker

## Abstract

Introduction: Hypertension is one of the most common cardiovascular diseases, and the prevalence of hypertension continues to rise across the globe. National and international guidelines recommend angiotensin-converting enzyme (ACE) inhibitors or angiotensin receptor blockers (ARBs), calcium channel blockers (CCBs), diuretics, and beta-blockers for the management of hypertension. CCBs are among the most used antihypertensive medications and Cilnidipine is a newer dihydropyridine CCB shown to have a prolonged antihypertensive property.

Objective: This meta-analysis of comparative randomized and non-randomized clinical trials evaluated the effect of Cilnidipine monotherapy or combination therapy on systolic blood pressure (SBP), diastolic blood pressure (DBP), and pulse rate (PR) over 48 weeks of therapy.

Study design: PubMed (MEDLINE) and Google scholar databases were searched to identify studies designed to evaluate the effects of Cilnidipine in the treatment of hypertensive patients. The study criteria for inclusion into the meta-analysis were all prospective, randomized, and non-randomized clinical studies published till March 2021, studies published in a peer-reviewed journal, the inclusion of patients with hypertension, assessment of blood pressure and heart rate, and a follow-up of four weeks or longer. The initial search identified 82 potential articles; of these, 24 met the inclusion criteria. Studies with <4 weeks treatment period and those not having a CCB were excluded.

Outcomes: Change in SBP, DBP, and PR from baseline at the end of therapy compared between the Cilnidipine and other CCB's.

Results: Cilnidipine caused a significant reduction (p<0.05) in SBP, DBP, and PR at end of therapy, whereas the reduction in SBP, DBP, and PR with Cilnidipine was similar to other CCB's (p>0.05). The results of this meta-analysis revealed that there were no significant differences in the efficacy in the treatment of hypertensive patients with Cilnidipine and the other therapies.

Conclusion: Cilnidipine has similar anti-hypertensive effects compared with other first-line antihypertensive drugs commonly used in practice. We recommend Cilnidipine as a novel first-line CCB for the management of hypertension either as a monotherapy or as a combination therapy.

## Introduction and background

Hypertension (HTN) is one of the most common cardiovascular diseases, and the prevalence of hypertension continues to rise across the globe [1]. Despite being so common, the awareness, treatment, and control of hypertension in the community are very less [1].

National and international guidelines recommend angiotensin-converting enzyme (ACE) inhibitors or angiotensin receptor blockers (ARBs), calcium channel blockers (CCBs), diuretics, and beta-blockers for the management of hypertension. CCBs are among the most used antihypertensive medications currently in the market, and the use of CCBs is especially effective for the treatment of hypertension in the elderly who frequently have large-vessel stiffness [2].

Calcium antagonists dilate blood vessels to reduce peripheral vascular resistance (PVR) which reduces blood pressure. The calcium blockers block calcium influx into vascular smooth muscle cells, resulting in vasodilatation and reduction of peripheral vascular resistance.

Cilnidipine is a newer dihydropyridine calcium antagonist shown to have a prolonged antihypertensive property [3]. Cilnidipine was first approved in Japan in 1995 and was subsequently approved by other countries to become one of the primary anti-hypertensive drugs used today.

Cilnidipine is an L/N-type calcium channel blocker, which lowers the BP in part by sympathetic nerve inhibition at the peripheral sympathetic nerve endings in vivo [4]. It has been shown to reduce both systolic blood pressure (SBP) and diastolic blood pressure (DBP) but does not increase pulse rates (PR) or plasma catecholamines [5]. It has also been shown to inhibit the pressor response to the acute cold stress in spontaneously hypertensive rats (SHR) [6]. Cilnidipine was reported to be effective in hypertensive patients with morning HTN in which sympathetic nerve overactivity was potentially involved. In hypertensive patients with abnormal nocturnal BP, Cilnidipine was also shown to significantly lower the BP, especially during sleep when exaggerated activation of the sympathetic nerve occurs [7]. Cilnidipine also attenuates vascular endothelial dysfunction and thus is useful in the long-term management of cardiovascular disorders [8]. The anti-hypertensive effects of Cilnidipine are significant, with good oral absorption and a long duration of action. After oral administration, drug concentrations peak at 1.8 to 2.2 hours and show a half-life of 7.5 hours. However, despite a shorter half-life, Cilnidipine exhibits a prolonged duration of anti-hypertensive action. It is postulated that Cilnidipine exhibits a high protein binding of 98%, which prolongs the duration of action. In-vitro and animal studies have shown that Cilnidipine action is slower in development and longer in duration compared to Nifedipine and Nicardipine [9,10].

Old CCB like Amlodipine and a newer CCB like Cilnidipine have shown equal efficacy in reducing blood pressure in hypertensive individuals. But Cilnidipine being an N‑type and L‑type calcium channel blocker is associated with a lower incidence of pedal edema compared to only the L‑type channel blocked by Amlodipine [11].

Cilnidipine has been reported to have more beneficial effects on proteinuria progression in hypertensive patients than Amlodipine, an L-type CCB [12]. The N-type calcium channel blockade that inhibits renal sympathetic nerve activity might reduce glomerular hypertension by facilitating vasodilation of the efferent arterioles. However, the precise mechanism of the renoprotective effect of Cilnidipine remains unknown. Because Cilnidipine exerted significantly higher antioxidant activity than Amlodipine in cultured human mesangial cells, it can be hypothesized that Cilnidipine might exert a renoprotective effect by suppressing oxidative stress. The urinary albumin, 8-hydroxy-2'-deoxyguanosine (OHdG), and liver-type fatty-acid-binding protein (L-FABP) to creatinine ratios significantly decreased with Cilnidipine (P<0.05) compared with those with Amlodipine [12]. Thus, Cilnidipine probably exerts a greater renoprotective effect through its antioxidative properties.

In a study conducted by Ramya et al., Cilnidipine was found to be safe and effective in reducing microalbuminuria and blood pressure in Indian mild-to-moderate hypertensive patients with type 2 diabetes mellitus. In this study, Cilnidipine caused a significant reduction in the mean (SD) SBP from 150.07 (5.44) mm Hg at baseline to 123.03 (5.23) mm Hg after six months. Cilnidipine also produced a significant reduction in the microalbuminuria from 66.62 (8.39) mg/L to 38.8 (6.45) mg/L after six months [13].

In another large-scale prospective post-marketing surveillance study of post-stroke hypertensive patients (n = 2667, male 60.4%, 69.0 ± 10.9 years) who were treated with Cilnidipine, the blood pressure control with Cilnidipine treatment was very good [14].

Although several meta-analyses have been published on the use of CCBs in cardiovascular disorders, only one has been reported with Cilnidipine [15]. This meta-analysis was conducted only on 11 randomized trials. There was a need for a robust meta-analysis to appraise the efficacy and safety of Cilnidipine in hypertensive patients. As a result, we performed a meta-analysis of comparative clinical trials (randomized and non-randomized) where Cilnidipine was used as monotherapy or combination therapy in the management of hypertension. We evaluated the effect of Cilnidipine on SBP, DBP, and PR over 48 weeks of therapy. We also performed a sub-group analysis based on the type of study (randomized controlled trial [RCT] versus non-RCT), use of ambulatory blood pressure monitoring (ABPM), presence or absence of diabetes mellitus (DM), and duration of therapy (4, 8, 12, 16, 24, and 48 weeks).

## Review

Review methodology

We performed a meta-analysis of comparative clinical trials (randomized and non-randomized) where Cilnidipine was used as monotherapy or combination therapy in the management of hypertension. This meta-analysis complied with the QUOROM (Quality of Reporting of Meta-analyses) statement [16]. PubMed (MEDLINE) and Google scholar databases were searched to identify studies designed to evaluate the effects of Cilnidipine in the treatment of hypertensive patients.

The study criteria for inclusion into the meta-analysis were all prospective, randomized, and non-randomized clinical studies published till March 2021, studies published in a peer-reviewed journal, the inclusion of patients with hypertension, assessment of blood pressure and heart rate, and a follow-up of four weeks or longer. Studies of all sample sizes were included. The search strategy was based on the search terms “Cilnidipine,” “Calcium Channel Blocker,” “CCB,” “Hypertension,” “Systolic Blood Pressure (SBP),” “Diastolic Blood Pressure (DBP),” “Heart rate,” “Pulse rate,” and “Ambulatory Blood Pressure Monitoring (ABPM).”

Abstracts of studies published in all languages (with English translations) were reviewed, and the full text was reviewed after the study satisfied the inclusion criteria. We excluded studies that were not available in English (translations), and studies that excluded hypertensive subjects.

**Figure 1:**
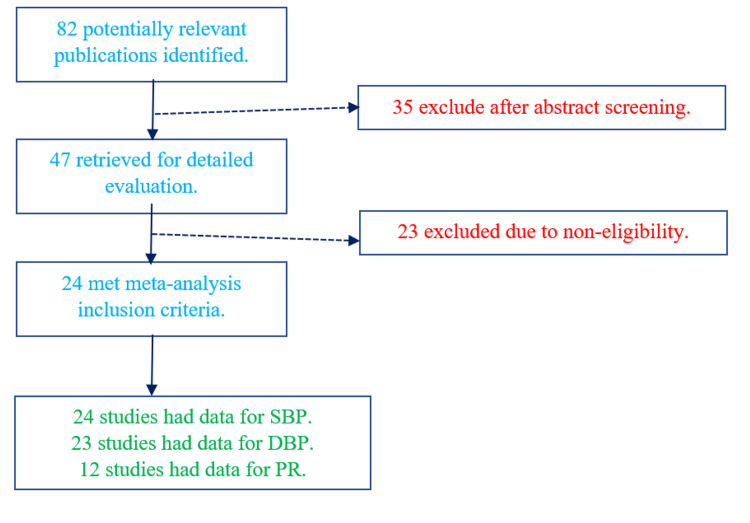
PRISMA 2009 study search and selection diagram PRISMA: Preferred Reporting Items for Systemic Reviews and Meta-analyses

The initial search identified 82 potential articles; of these, 24 met the inclusion criteria (Figure 1). One study was published as a post-graduate dissertation. The details of the studies included for meta-analysis are listed in Table 1.

**Table 1:** Characteristics of studies included for meta-analysis RCT: randomized controlled trial

Study	Design	BP measurement	Study patients	FDC	N (Cilnidipine)	N (Control)	Control	Treatment duration (weeks)
Minami et al. [17]	RCT	Ambulatory	Non-diabetic	No	5	5	Other	8
Sakata et al. [18]	RCT	Office	Non-diabetic	No	23	24	Amlodipine	12
Takeda et al. [19]	RCT	Office	Diabetic	No	35	35	Other	4
Rose et al. [20]	RCT	Office	Non-diabetic	No	10	10	Other	48
Kojima et al. [21]	RCT	Office	Non-diabetic	No	14	14	Amlodipine	24
Tsuchihashi et al. [22]	RCT	Office	Non-diabetic	No	25	18	Other	24
Morimoto et al. [23]	RCT	Office	Non-diabetic	No	25	25	Amlodipine	24
Fujita et al. [24]	RCT	Office	Non-diabetic	No	179	160	Amlodipine	48
Hong et al. [25]	RCT	Office	Non-diabetic	No	98	98	Other	4
Konoshita et al. [26]	RCT	Office	Non-diabetic	No	55	55	Amlodipine	12
Abe et al. [27]	RCT	Office	Non-diabetic	No	115	118	Other	48
Miwa et al. [28]	RCT	Office	Non-diabetic	No	18	17	Amlodipine	48
Abe et al. [29]	RCT	Office	Non-diabetic	No	35	35	Amlodipine	48
Kanoaka et al. [30]	RCT	Ambulatory	Non-diabetic	No	21	24	Amlodipine	24
Adake et al. [11]	Non-RCT	Office	Non-diabetic	No	30	30	Amlodipine	12
Singh et al. [31]	RCT	Office	Diabetic	Yes	35	36	Other	48
Pathapati et al. [32]	RCT	Office	Non-diabetic	No	30	30	Amlodipine	8
Masaki et al. [33]	RCT	Office	Non-diabetic	No	31	31	Amlodipine	48
Das et al. [34]	RCT	Office	Non-diabetic	No	45	47	Amlodipine	24
Singal et al. [35]	Non-RCT	Office	Non-diabetic	No	50	50	Amlodipine	24
Hwang et al. [36]	RCT	Office	Diabetic	No	38	36	Amlodipine	24
Oh et al. [37]	Non-RCT	Office	Non-diabetic	No	28	25	Other	48
Kawabata et al. [38]	Non-RCT	Office	Non-diabetic	Yes	63	66	Other	8
Fujiwara et al. [39]	Non-RCT	Office	Non-diabetic	No	12	13	Amlodipine	24

Outcomes

The data were abstracted, and differences were re­solved by consensus. Data of change in various parameters from baseline to end of therapy were estimated using standard formulae based on analysis of paired data. Efficacy outcomes included SBP, DBP, and PR. Of the twenty-four studies, two studies present blood pressure using a 24-hour ambulatory recording. For these studies, the daytime values for SBP, DBP, and PR were used for analysis along with other studies' data. There were 18 RCTs and six non-RCTs, three studies had diabetic hypertensive patients, two used fixed-dose therapy of Cilnidipine with some other antihypertensive drug, and 15 studies had Amlodipine as control therapy.

Statistical analyses

Meta-analysis was performed using windows based ‘MedCalc Statistical Software’ version 19.6.1 (2020). Data computations and imputations did in Stata-IC 13.1 (StataCorp LLC, College Station, TX, USA). A meta-analysis was done using the random-effects model for comparisons baseline (pre-treatment) versus post-treatment values for different measurement parameters (continuous measure). The random-effects model tends to give a more conservative estimate (i.e., with a wider confidence interval), but the results from the two models usually agree where there is no heterogeneity. Since the current data are heterogenous (p<0.05), the random-effects model was used. The standardized mean differences (SMD, effect size) were calculated for SBP, DBP, and PR using the Hedges g statistic, which is the difference between the two means (Cilnidipine and control) divided by the pooled standard deviation (SD), with a correction for small sample bias. If the value 0 was not inside the 95% CI, then the SMD is statistically significant at the 5% level (P<0.05). Cohen's rule of thumb for interpretation of the SMD statistic is: a value of 0.2 indicates a small effect, a value of 0.5 indicates a medium effect, and a value of 0.8 or more indicates a large effect. Publication bias was estimated using the funnel plot. Heterogeneity of the data is estimated using Cohran's Q and I2 statistics, where I2 values ≥50% were indicators of a substantial level of heterogeneity. Forest plots were also used for visual inspection. Funnel plots of effect estimates against the standard error were examined to assess publication bias. Sub-group analysis was done for all parameters based on study design (RCT and non-RCT), diabetes status, a therapy used (fixed-dose combination therapy or monotherapy), setting of blood pressure recording (ambulatory and office setting), duration of therapy (4, 8, 12, 16, 24, and 48 weeks), and comparator used (Amlodipine and other drugs).

Systolic blood pressure

**Figure 2:**
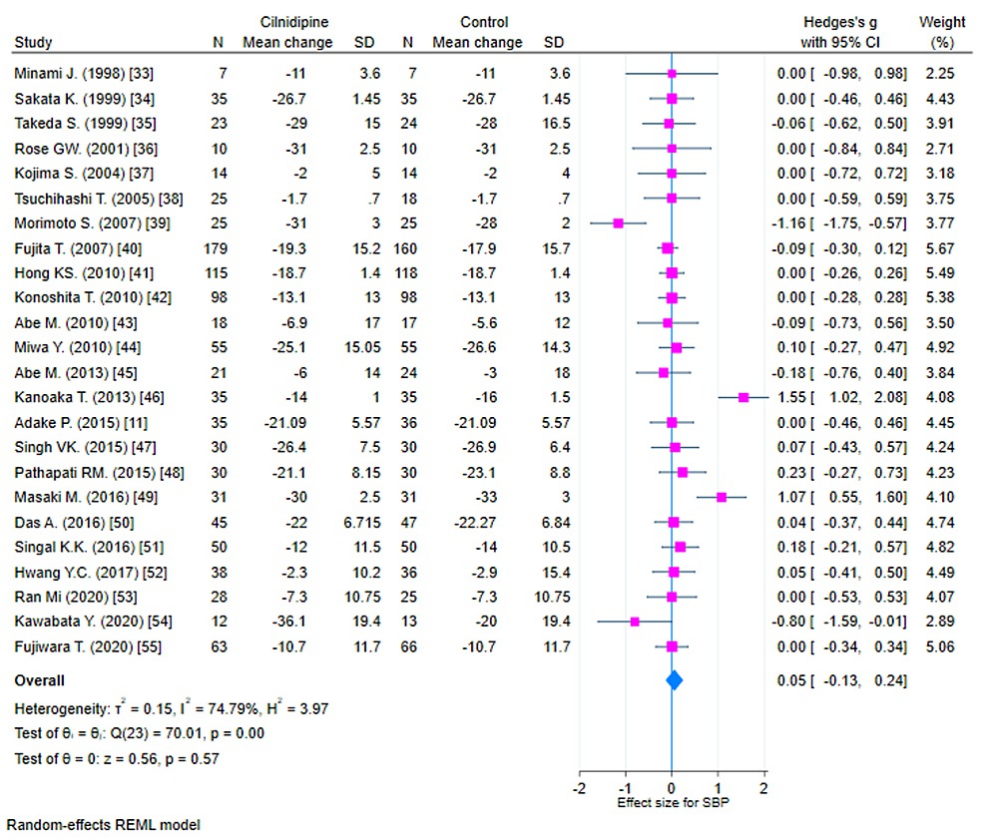
Forest plot for change in SBP (K=24) SBP: systolic blood pressure; REML: random-effects model; SD: standard deviation; CI: confidence intervals Minami et al. [17]; Sakata et al. [18]; Takeda et al. [19]; Rose et al. [20]; Kojima et al. [21]; Tsuchihashi et al. [22]; Morimoto et al. [23]; Fujita et al. [24]; Hong et al. [25]; Konoshita et al. [26]; Abe et al. [27]; Miwa et al. [28]; Abe et al. [29]; Kanoaka et al. [30]; Adake et al. [11]; Singh et al. [31]; Pathapati et al. [32]; Masaki et al. [33]; Das et al. [34]; Singal et al. [35]; Hwang et al. [36]; Oh et al. [37]; Kawabata et al. [38]; Fujiwara et al. [39]

**Figure 3:**
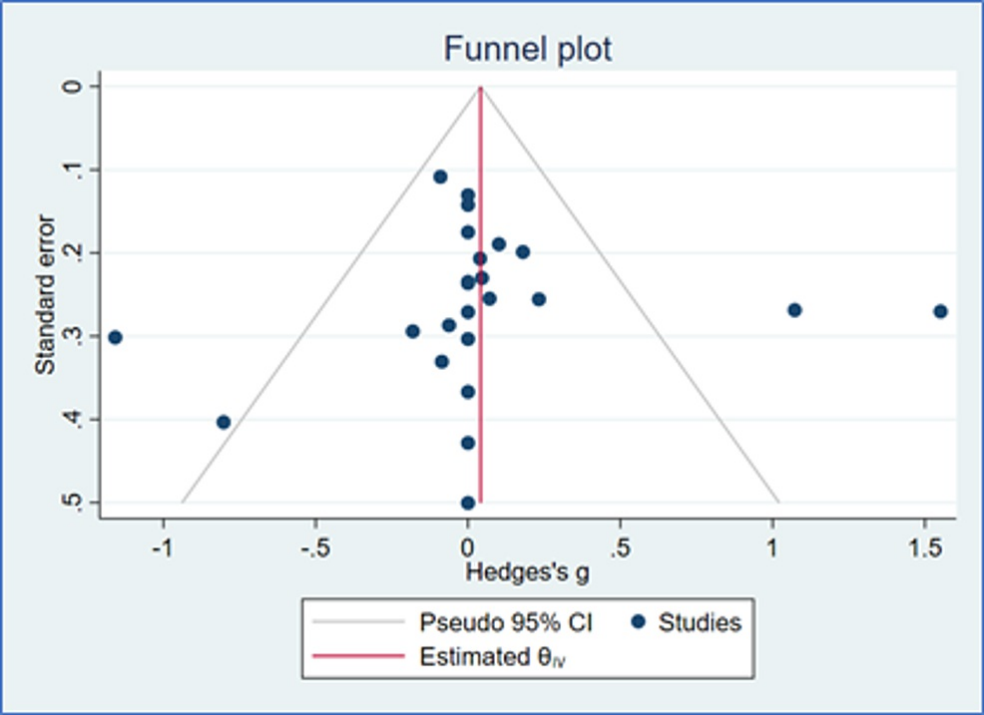
Funnel plot for SBP (K=24) CI: confidence intervals

**Figure 4:**
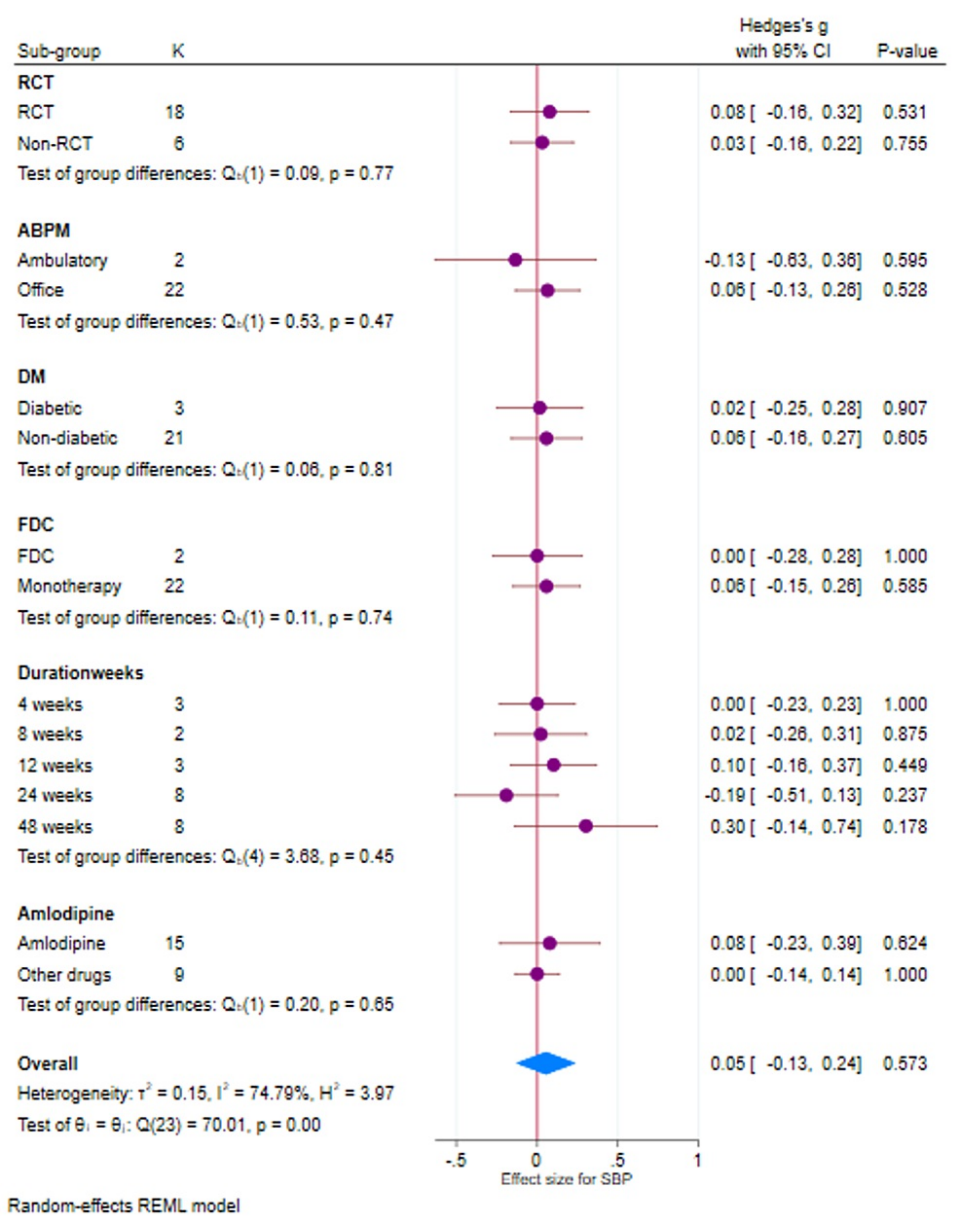
Forest plot (sub-groups) for SBP (K=24) SBP: systolic blood pressure; REML: random-effects model; SD: standard deviation; CI: confidence intervals; ABPM: ambulatory blood pressure monitoring; FDC: fixed-dose combination; DM: diabetes mellitus

There were 24 studies that had evaluable data for SBP. There was a reduction in SBP with both Cilnidipine-based therapy and control (Amlodipine and non-Amlodipine-based therapy) in all patients with hypertension (Figure 2). The overall effect size (Hedge’s “g”) for differences between Cilnidipine and control therapy for change in SBP was 0.05 (95% CI, −0.13 to 0.24; p=0.573). Figure 3 presents the funnel plot for change in SBP. There were four studies that had publication bias. Figure 4 presents the forest plot for change in SBP in different sub-groups. There were no differences in the different sub-groups (p>0.05) with respect to the reduction in SBP.

Diastolic blood pressure

**Figure 5:**
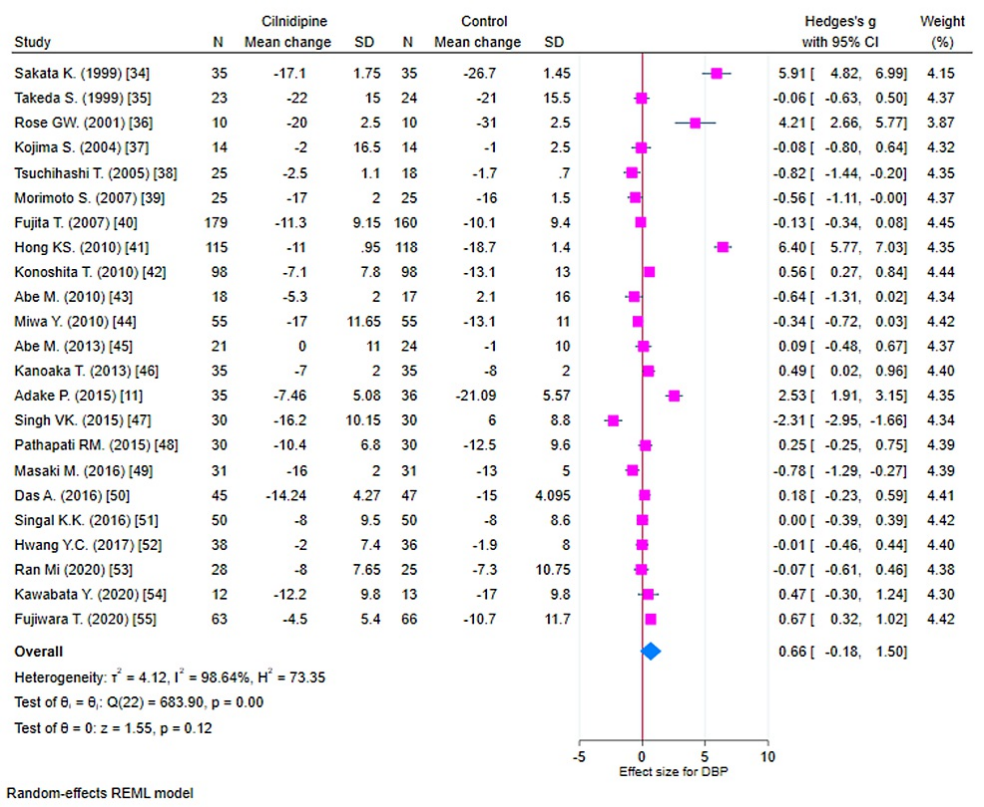
Forest plot for change in DBP (K=23) DBP: diastolic blood pressure; REML: random-effects model; SD: standard deviation: CI: confidence intervals Sakata et al. [18]; Takeda et al. [19]; Rose et al. [20]; Kojima et al. [21]; Tsuchihashi et al. [22]; Morimoto et al. [23]; Fujita et al. [24]; Hong et al. [25]; Konoshita et al. [26]; Abe et al. [27]; Miwa et al. [28]; Abe et al. [29]; Kanoaka et al. [30]; Adake et al. [11]; Singh et al. [31]; Pathapati et al. [32]; Masaki et al. [33]; Das et al. [34]; Singal et al. [35]; Hwang et al. [36]; Oh et al. [37]; Kawabata et al. [38]; Fujiwara et al. [39]

**Figure 6:**
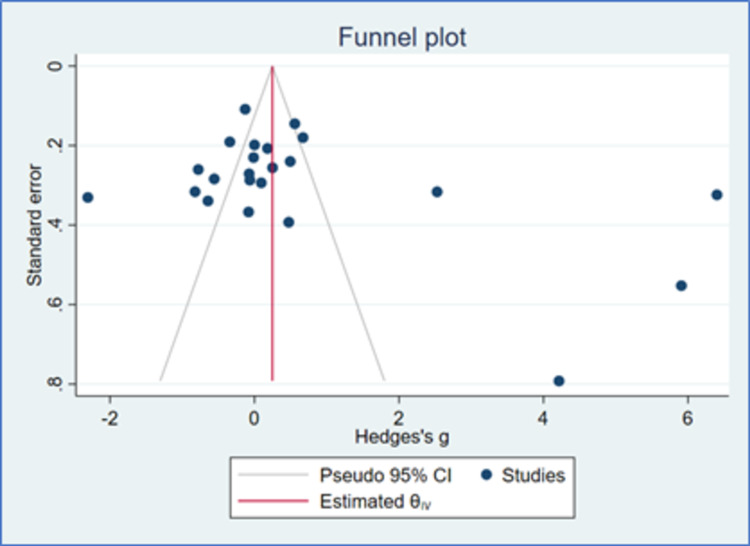
Funnel plot for DBP (K=23) CI: confidence intervals, DBP: diastolic blood pressure

**Figure 7:**
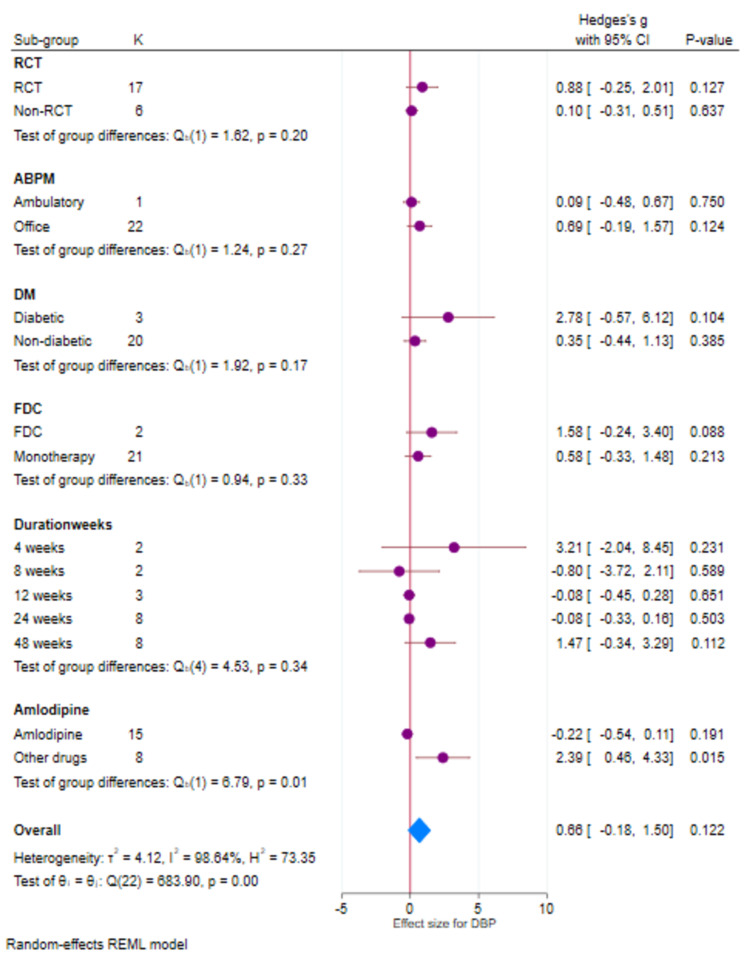
Forest plot (sub-groups) for DBP (K=12) DBP: diastolic blood pressure; REML: random-effects model; SD: standard deviation; CI: confidence intervals; ABPM: ambulatory blood pressure monitoring; FDC: fixed-dose combination; DM: diabetes mellitus

There were 23 studies that had evaluable data for DBP. There was a reduction in DBP with both Cilnidipine-based therapy and control (Amlodipine and non-Amlodipine-based therapy) in all patients with hypertension (Figure 5). The overall effect size (Hedge’s “g”) for differences between Cilnidipine and control therapy for change in DBP was 0.66 (95% CI, −0.18 to 1.50; p=0.122). Figure 6 presents the funnel plot for change in DBP. There were 13 studies that had publication bias. Figure 7 presents the forest plot for change in DBP in different sub-groups. There were significant differences (p=0.01) between Cilnidipine and control therapy with respect to the control used (Amlodipine and non-Amlodipine-based therapy), where Amlodipine-based therapy had lesser reduction (p=0.191, effect size −0.22, 95% CI −0.54 to 0.11) in DBP compared to other therapies (p=0.015, effect size 2.39, 95% CI 0.46 to 4.33). There were no differences in the other sub-groups (p>0.05) with respect to the reduction in DBP.

Pulse rate

**Figure 8:**
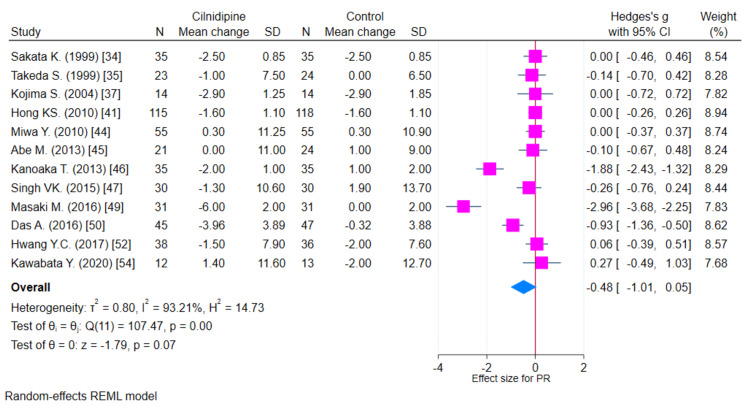
Forest plot for change in PR (K=12) PR: pulse rate; REML: random-effects model; SD: standard deviation: CI: confidence intervals Sakata et al. [18]; Takeda et al. [19]; Kojima et al. [21]; Hong et al. [25]; Miwa et al. [28]; Abe et al. [29]; Kanoaka et al. [30]; Adake et al. [11]; Singh et al. [31]; Masaki et al. [33]; Das et al. [34]; Hwang et al. [36]; Kawabata et al. [38]

**Figure 9:**
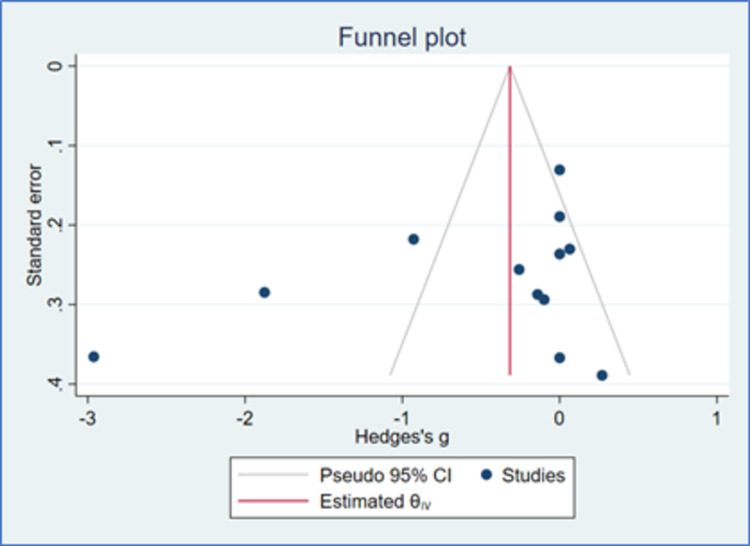
Funnel plot for PR (K=12) CI: confidence intervals

**Figure 10:**
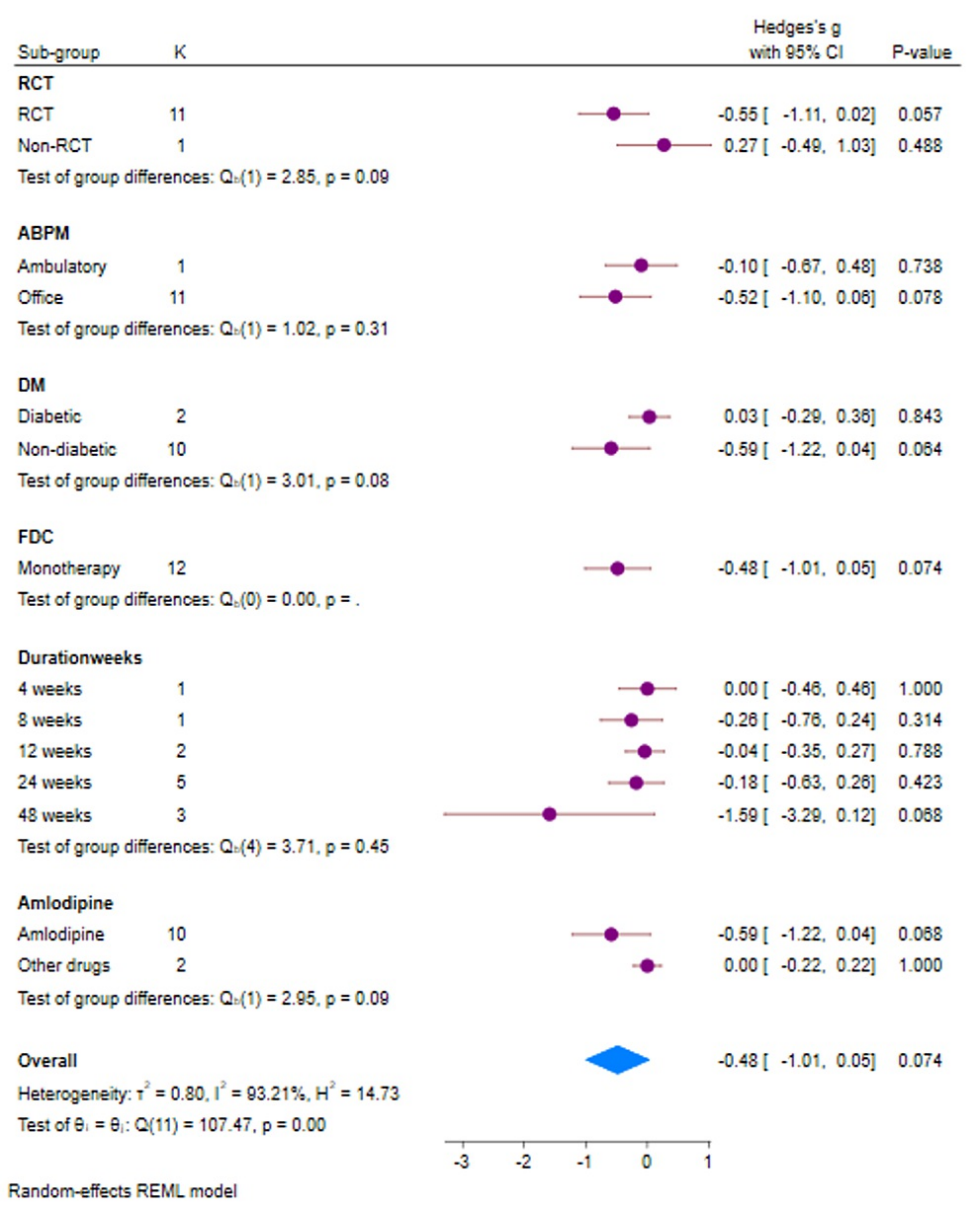
Forest plot (sub-groups) for PR (K=12) PR: pulse rate; REML: random-effects model; SD: standard deviation: CI: confidence intervals; ABPM: ambulatory blood pressure monitoring; FDC: fixed-dose combination; DM: diabetes mellitus

There were only 12 studies that provided data of PR which could be analyzed. There was a reduction in PR with both Cilnidipine-based therapy and control (Amlodipine and non-Amlodipine-based therapy) in all patients with hypertension (Figure 8). The overall effect size (Hedge’s “g”) for differences between Cilnidipine and control therapy for change in PR was −0.48 (95% CI, −1.01 to 0.05; p=0.074). Figure 9 presents the funnel plot for change in PR. There were three studies that had publication bias. Figure 10 presents the forest plot for change in PR in different sub-groups. There were no differences in any of the sub-groups (p>0.05) with respect to the reduction in PR.

Sensitivity analyses

In the efficacy analysis, there was no difference in the overall response rates between Cilnidipine and the control group for all efficacy parameters assessed. The effect size varied from −0.48 (PR) to 0.66 (DBP) without any statistical significance (p>0.05). Further, no differences were found in the different sub-groups based on study design, diabetes status, therapy used, setting of blood pressure recording, duration of therapy, and comparator used.

Summary of the literature quality

In an analysis of the articles, we found that all trials that were included in the meta-analysis were of high quality. The Jadad score was at least two points for each of the 24 studies used for the meta-analysis. There was evidence of publication bias found for SBP (four studies), DBP (13 studies), and PR (three studies). However, there were significant heterogeneities between studies for SBP (I2=74.79%, p<0.0001), DBP (I2=98.64%, p<0.0001), and PR (I2=93.21%, p<0.0001). Combined, this suggests that the overall quality of the analysis was moderate to good.

Discussion

The current meta-analysis showed that Cilnidipine significantly reduced systolic blood pressure, diastolic blood pressure, and pulse rate in hypertensive patients. Cilnidipine has been extensively studied in the management of hypertension. Cilnidipine has been proved to have a reno, neuro, and cardioprotective effect. It decreases heart rate and proteinuria, apart from its BP-lowering effect [13]. Hypertension is a leading cause of cardiovascular morbidity and mortality and congestive heart failure (CHF) due to the increased work overload on the myocardium [15]. Despite several initiatives, the prevalence of raised BP and adverse impact on cardiovascular morbidity and mortality are increasing globally, irrespective of income [40]. The International Society of Hypertension (ISH) issued guidelines in 2020 on the management of hypertension and recommends prompt control of blood pressure to a goal of less than 140 mmHg systolic and 90 mmHg diastolic blood pressure [41]. The ISH and other guidelines from regions and countries, including the US [42], Europe [43], United Kingdom [44], Canada [45], and Japan [46] advise initiating treatment with single-pill combination therapy [42], greater out-of-office BP measurement [46], and lower BP targets [47]. First-line medications used in the treatment of hypertension include diuretics, ACE inhibitors or ARBs, beta-blockers, and CCBs [48]. Some patients may require two or more antihypertensive medications to achieve their BP target. Among these ARBs and CCBs are the preferred agents for hypertension management. CCBs provide benefits in reducing the development of CHF, angina, and renal complications. CCBs are most beneficial in diabetics with hypertension and may also provide protection for stroke [48]. CCBs lower BP by preventing the entry of calcium into vascular smooth muscles, resulting in vasodilation and reduced vascular contractility. The two types of CCBs are (1) dihydropyridines, which act on peripheral blood vessels, and (2) non-dihydropyridines, which act on cardiac muscles and peripheral blood vessels. Randomized controlled trials have demonstrated that dihydropyridines are effective at reducing CV events, mortality, and strokes particularly in the elderly [49]. Non-dihydropyridines are useful in the treatment of cardiac arrhythmias. Both types of drugs are effective as monotherapy in reducing BP and are generally well tolerated. Recent results from the ACCOMPLISH trial have shown that CCBs are comparable first-line agents and are well tolerated when combined with another drug, especially an ACE inhibitor [50]. JNC-7 recognizes CCBs as a possible first-line drug class for patients at high risk for CVD or for those with diabetes [51]. Amlodipine is one of the commonly used CCB for the treatment of cardiovascular diseases. However, these older CCBs are associated with adverse events majorly due to activation of the sympathetic nervous system. L-type CCBs are associated with certain limitations like limited organ protection properties and adverse events like reflex tachycardia and edema, common side effects that can affect compliance [52]. Cilnidipine is a relatively newer dihydropyridine CCB which is an L/N-type calcium channel blocker and has an additional action at the peripheral sympathetic nerve endings by sympathetic nerve inhibition [4]. It does not increase PR or plasma catecholamines [5]. In hypertensive patients with abnormal nocturnal BP, Cilnidipine was also shown to significantly lower the BP, especially during sleep when exaggerated activation of sympathetic nerve occurred [7]. Cilnidipine is a highly lipophilic dihydropyridine CCB. Cilnidipine shows high vascular selectivity, slow onset, and a longer duration of hypotensive action than the earlier generation CCBs. Cilnidipine exhibits stable anti-hypertensive activity and reduced adverse effects. It has been reported that Cilnidipine reduces excessive excitation of the sympathetic nervous system and the release of norepinephrine from sympathetic nerve endings, and consequently suppresses reflective tachycardia and stress-induced BP elevation, which is more efficient than Amlodipine. Cilnidipine also leads to less activation of the renin-angiotensin system than Amlodipine, and thus, is expected to play a superior role in organ protection [53]. Blood pressure variability is considered nowadays a novel risk factor for cardiovascular disease. Blood pressure variability correlates closely with target-organ damage independent of mean BP and transient increases in BP. The goals of antihypertensive treatment should consider the reduction of both 24-hour mean BP and its variability [54]. Nishioka et al. measured the 24-hour blood pressure variability in 309 patients with a history of cerebrovascular disease treated with angiotensin-converting enzyme inhibitor, angiotensin receptor blocker, beta-blockers, or a calcium channel blocker. Treatment with angiotensin receptor blockers and Cilnidipine was shown to be more frequently associated with lower blood pressure variability (P=0.0202 and P=0.0467). Among the calcium channel blockers, Cilnidipine showed low BP variability (P=0.0467) as compared to other CCBs. A higher proportion of patients administered Cilnidipine showed low BP variability whereas no relationship was noted in the patients administered other CCBs like Amlodipine, Nifedipine, and Nicardipine. It has been reported that the low BP variability obtained with CCB therapy is not dependent on the half-life of the drugs. From the above, it is suggested that among the CCBs, Cilnidipine may be particularly effective for reducing the risk of recurrent CVD [55]. This meta-analysis of comparative clinical trials (randomized and non-randomized) was conducted to evaluate the efficacy of Cilnidipine monotherapy or combination therapy in the management of hypertension. We evaluated the effect of Cilnidipine on SBP, DBP, and PR over 48 weeks of therapy. We also performed a sub-group analysis based on the type of study (RCT versus non-RCT), use of ABPM, presence or absence of DM, and duration of therapy (4, 8, 12, 16, 24, and 48 weeks). We observed that the efficacy of Cilnidipine is similar to the other antihypertensive drugs used as monotherapy or combination therapy in the management of hypertension. In the sub-group analysis, Cilnidipine was as good as Amlodipine in terms of efficacy and safety. In the current meta-analysis, Amlodipine-based therapy was found to have a lesser reduction (p=0.191, effect size −0.22, 95% CI −0.54 to 0.11) in DBP compared to other therapies (p=0.015, effect size 2.39, 95% CI 0.46 to 4.33). The current meta-analysis showed that Cilnidipine provides adequate blood pressure control at therapeutic doses in the management of hypertension and these effects are similar to other antihypertensive agents.

There were few methodological insufficiencies that require mention. These include (i) randomization methods for the individual studies may not be rigorous because the methods were not clearly described in a few studies; (ii) possibility of selection bias due to poorly described allocation concealment methods; (iii) possibility of measurement bias because the study design was not described in one study; and (iv) we did not evaluate the clinical and laboratory adverse events in the analysis.

## Conclusions

The current meta-analysis of 24 clinical trials showed that Cilnidipine significantly reduced systolic blood pressure, diastolic blood pressure, and pulse rate in hypertensive patients. The results of this meta-analysis revealed that there were no significant differences in the efficacy in the treatment of hypertensive patients with Cilnidipine and the other therapies. However, there is the possibility of selection bias in a few studies. We can conclude, therefore, that Cilnidipine has similar anti-hypertensive effects compared with other first-line antihypertensive drugs commonly used in practice.

We recommend Cilnidipine as a novel first-line CCB for the management of hypertension either as a monotherapy or as a combination therapy. Cilnidipine is highly lipophilic and shows low BP variability among CCBs. The organ protection, especially the reno-protective effect of Cilnidipine deserves special attention. In earlier trials, Cilnidipine was found to be safe and effective in reducing microalbuminuria in hypertensive patients. We warrant further studies to reinforce the cardio-protection and renoprotection efficacy of Cilnidipine, particularly in hypertensive diabetic patients.

## References

[1] (2019). 2018 Chinese Guidelines for prevention and treatment of hypertension: A report of the Revision Committee of Chinese Guidelines for Prevention and Treatment of Hypertension. J Geriatr Cardiol.

[2] Iyer RP, Lindsey ML, Chilton RJ (2013). A two-for-one bargain: using cilnidipine to treat hypertension and its comorbidities. J Clin Hypertens (Greenwich).

[3] Ikeda K, Hosino M, Iida H, Ohnishi H (1992). Hypertensive and cardiovascular profiles of a newly synthesized dihydropyridine derivative 2-methoxyethyl (E)-3-phenyl-2-propen-1-yl(6)-1,4-dihydro-2,6-dimethyl-4-(3-nitrophenyl) pyridine-3,5-dicarboxylate (FRC-8653). Pharmacometrics.

[4] Kishi T, Hirooka Y, Konno S, Sunagawa K (2009). Cilnidipine inhibits the sympathetic nerve activity and improves baroreflex sensitivity in patients with hypertension. Clin Exp Hypertens.

[5] Hosono M, Hiruma T, Watanabe K, Hayashi Y, Ohnishi H, Takata Y, Kato H (1995). Inhibitory effect of cilnidipine on pressor response to acute cold stress in spontaneously hypertensive rats. Jpn J Pharmacol.

[6] Hiruma T, Hosono M, Watanabe K, Hayashi Y, Ohnishi H (1995). Changes in heart rate and plasma catecholamine levels accompanied with hypotesive action of calcium channel blockers in conscious SHR. Jpn J Pharmacol.

[7] Kario K, Ando S, Kido H (2013). The effects of the L/N-type calcium channel blocker (cilnidipine) on sympathetic hyperactive morning hypertension: results from ACHIEVE-ONE. J Clin Hypertens (Greenwich).

[8] Fan L, Yang Q, Xiao XQ (2011). Dual actions of cilnidipine in human internal thoracic artery: inhibition of calcium channels and enhancement of endothelial nitric oxide synthase. J Thorac Cardiovasc Surg.

[9] Yoshimoto R, Dohmoto H, Yamada K, Goto A (1991). Prolonged inhibition of vascular contraction and calcium influx by the novel 1,4-dihydropyridine calcium antagonist cinaldipine (FRC-8653). Jpn J Pharmacol.

[10] Hosono M, Iida H, Ikeda K (1992). In vitro and ex vivo Ca-antagonistic effect of 2-methoxyethyl(E)-3-phenyl-2-propen-1-yl(+/-)-1,4-dihydro-2,6-dimethyl- 4-(3- nitrophenyl)pyridine-3,5-dicarboxylate (FRC-8653), a new dihydropyridine derivative. J Pharmacobiodyn.

[11] Adake P, Somashekar HS, Mohammed Rafeeq PK, Umar D, Basheer B, Baroudi K (2015). Comparison of amlodipine with cilnidipine on antihypertensive efficacy and incidence of pedal edema in mild to moderate hypertensive individuals: A prospective study. J Adv Pharm Technol Res.

[12] Soeki T, Kitani M, Kusunose K (2012). Renoprotective and antioxidant effects of cilnidipine in hypertensive patients. Hypertens Res.

[13] Ramya R, Shahan OM, Anakha K (2021). Evaluation of renoprotective effect of cilnidipine in patients with mild to moderate hypertension and type 2 diabetes mellitus - a prospective study. Asian J Pharm Clin Res.

[14] Aoki S, Hosomi N, Nezu T (2017). Blood pressure control with cilnidipine treatment in Japanese post-stroke hypertensive patients: the CA-ATTEND study. Clin Exp Hypertens.

[15] Xu G, Wu H, Du B, Qin L (2012). The efficacy and safety of cilnidipine on mild to moderate essential hypertension: a systematic review and meta-analysis of randomized controlled trials in Chinese patients. Cardiovasc Hematol Disord Drug Targets.

[16] Moher D, Cook DJ, Eastwood S, Olkin I, Rennie D, Stroup DF (1999). Improving the quality of reports of meta-analyses of randomised controlled trials: the QUOROM statement. Lancet.

[17] Minami J, Ishimitsu T, Higashi T, Numabe A, Matsuoka H (1998). Comparison between cilnidipine and nisoldipine with respect to effects on blood pressure and heart rate in hypertensive patients. Hypertens Res.

[18] Sakata K, Shirotani M, Yoshida H, Nawada R, Obayashi K, Togi K, Miho N (1999). Effects of amlodipine and cilnidipine on cardiac sympathetic nervous system and neurohormonal status in essential hypertension. Hypertension.

[19] Takeda S, Ueshiba H, Hattori Y (1999). Cilnidipine, the N- and L-type calcium channel antagonist, reduced on 24-h urinary catecholamines and C-peptide in hypertensive non-insulin-dependent diabetes mellitus. Diabetes Res Clin Pract.

[20] Rose GW, Kanno Y, Ikebukuro H (2001). Cilnidipine is as effective as benazepril for control of blood pressure and proteinuria in hypertensive patients with benign nephrosclerosis. Hypertens Res.

[21] Kojima S, Shida M, Yokoyama H (2004). Comparison between cilnidipine and amlodipine besilate with respect to proteinuria in hypertensive patients with renal diseases. Hypertens Res.

[22] Tsuchihashi T, Ueno M, Tominaga M, Kajioka T, Onaka U, Eto K, Goto K (2005). Anti-proteinuric effect of an N-type calcium channel blocker, cilnidipine. Clin Exp Hypertens.

[23] Morimoto S, Yano Y, Maki K, Iwasaka T (2007). Renal and vascular protective effects of cilnidipine in patients with essential hypertension. J Hypertens.

[24] Fujita T, Ando K, Nishimura H, Ideura T, Yasuda G, Isshiki M, Takahashi K (2007). Antiproteinuric effect of the calcium channel blocker cilnidipine added to renin-angiotensin inhibition in hypertensive patients with chronic renal disease. Kidney Int.

[25] Hong KS, Kang DW, Bae HJ (2010). Effect of cilnidipine vs losartan on cerebral blood flow in hypertensive patients with a history of ischemic stroke: a randomized controlled trial. Acta Neurol Scand.

[26] Konoshita T, Makino Y, Kimura T (2010). A new-generation N/L-type calcium channel blocker leads to less activation of the renin-angiotensin system compared with conventional L type calcium channel blocker. J Hypertens.

[27] Abe M, Okada K, Maruyama T, Maruyama N, Matsumoto K (2009). Comparison of the antiproteinuric effects of the calcium channel blockers benidipine and amlodipine administered in combination with angiotensin receptor blockers to hypertensive patients with stage 3-5 chronic kidney disease. Hypertens Res.

[28] Miwa Y, Tsuchihashi T, Ohta Y (2010). Antiproteinuric effect of cilnidipine in hypertensive Japanese treated with renin-angiotensin-system inhibitors - a multicenter, open, randomized trial using 24-hour urine collection. Clin Exp Hypertens.

[29] Abe M, Maruyama N, Suzuki H, Inoshita A, Yoshida Y, Okada K, Soma M (2013). L/N-type calcium channel blocker cilnidipine reduces plasma aldosterone, albuminuria, and urinary liver-type fatty acid binding protein in patients with chronic kidney disease. Heart Vessels.

[30] Kanaoka T, Tamura K, Wakui H (2013). L/N-type calcium channel blocker cilnidipine added to renin-angiotensin inhibition improves ambulatory blood pressure profile and suppresses cardiac hypertrophy in hypertension with chronic kidney disease. Int J Mol Sci.

[31] Singh VK, Mishra A, Gupta KK, Misra R, Patel ML, Shilpa Shilpa (2015). Reduction of microalbuminuria in type-2 diabetes mellitus with angiotensin-converting enzyme inhibitor alone and with cilnidipine. Indian J Nephrol.

[32] Pathapati RM, Rajashekar ST, Buchineni M, Meriga RK, Reddy CB, Kumar KP (2015). An open label parallel group study to assess the effects of amlodipine and cilnidipine on pulse wave velocity and augmentation pressures in mild to moderate essential hypertensive patients. J Clin Diagn Res.

[33] Masaki M, Mano T, Eguchi A (2016). Long-term effects of L- and N-type calcium channel blocker on uric acid levels and left atrial volume in hypertensive patients. Heart Vessels.

[34] Das A, Kumar P, Kumari A, Chandra S, Gari M, Singh N, Dey D (2016). Effects of cilnidipine on heart rate and uric acid metabolism in patients with essential hypertension. Cardiol Res.

[35] Singal KK, Singal N, Gupta A (2016). Comparison of the effects of L-type calcium channel antagonist Amlodipine with L/N-type calcium channel antagonist Cilnidipine on blood pressure, heart rate, proteinuria and lipid profile in hypertensive patients. Bangladesh J Med Sci.

[36] Hwang YC, Yoon KH, Cha BS (2017). Reduction in microalbuminuria by calcium channel blockers in patients with type 2 diabetes mellitus and hypertension-A randomized, open-label, active-controlled, superiority, parallel-group clinical trial. Int J Clin Pract.

[37] Oh MR, Ahn HL, Choi S, La HO (2020). Comparison of usage patterns and outcomes by dual type calcium channel blockers in patients with chronic kidney disease. Korean J Clin Pharm.

[38] Kawabata Y, Soeki T, Ito H (2020). Effects of L-/N-type calcium channel blockers on angiotensin II-renin feedback in hypertensive patients. Int J Hypertens.

[39] Fujiwara T, Hoshide S, Tomitani N (2021). Clinical significance of nocturnal home blood pressure monitoring and nocturnal hypertension in Asia. J Clin Hypertens (Greenwich).

[40] (2017). Worldwide trends in blood pressure from 1975 to 2015: a pooled analysis of 1479 population-based measurement studies with 19·1 million participants. Lancet.

[41] Unger T, Borghi C, Charchar F (2020). 2020 International Society of Hypertension Global Hypertension Practice Guidelines. Hypertension.

[42] Whelton PK, Carey RM, Aronow WS (2018). 2017 ACC/AHA/AAPA/ABC/ACPM/AGS/APhA/ASH/ASPC/NMA/PCNA Guideline for the prevention, detection, evaluation, and management of high blood pressure in adults: a report of the American College of Cardiology/American Heart Association Task Force on Clinical Practice Guidelines. Hypertension.

[43] Williams B, Mancia G, Spiering W (2018). 2018 ESC/ESH guidelines for the management of arterial hypertension. Eur Heart J.

[44] (2021). Hypertension in adults: diagnosis and management. https://www.nice.org.uk/guidance/ng136.

[45] Nerenberg KA, Zarnke KB, Leung AA (2018). Hypertension Canada's 2018 guidelines for diagnosis, risk assessment, prevention, and treatment of hypertension in adults and children. Can J Cardiol.

[46] Umemura S, Arima H, Arima S (2019). The Japanese Society of Hypertension Guidelines for the management of hypertension (JSH 2019). Hypertens Res.

[47] Kario K, Wang JG (2018). Could 130/80 mm Hg Be Adopted as the Diagnostic Threshold and Management Goal of Hypertension in Consideration of the Characteristics of Asian Populations?. Hypertension.

[48] Nguyen Q, Dominguez J, Nguyen L, Gullapalli N (2010). Hypertension management: an update. Am Health Drug Benefits.

[49] Neal B, MacMahon S, Chapman N; Blood Pressure Lowering Treatment Trialists' Collaboration (2000). Effects of ACE inhibitors, calcium antagonists, and other blood pressure-lowering drugs results of prospectively designed overviews of randomised trials. Lancet.

[50] Jamerson K, Weber MA, Bakris GL (2008). Benazepril plus amlodipine or hydrochlorothiazide for hypertension in high-risk patients. N Engl J Med.

[51] Chobanian AV, Bakris GL, Black HR (2003). Seventh report of the Joint National Committee on Prevention, Detection, Evaluation, and Treatment of High Blood Pressure. Hypertension.

[52] Ozawa Y, Hayashi K, Kobori H (2006). New generation calcium channel blockers in hypertensive treatment. Curr Hypertens Rev.

[53] Wang AL, Iadecola C, Wang G (2017). New generations of dihydropyridines for treatment of hypertension. J Geriatr Cardiol.

[54] Choi HJ (2012). Blood pressure variability and its management in hypertensive patients. Korean J Fam Med.

[55] Nishioka R, Kinoshita S, Shiibashi M (2015). Evaluation of the differences in the effects of antihypertensive drugs on blood pressure variability by 24-hour ambulatory blood pressure monitoring in chronic cerebrovascular disease. J Stroke Cerebrovasc Dis.

